# Nanopore sensing and machine learning: future of biomarker analysis and disease detection

**DOI:** 10.2144/fsoa-2023-0226

**Published:** 2024-05-15

**Authors:** Shankar Dutt, Buddini Karawdeniya, Yapa MNDY Bandara, Patrick Kluth

**Affiliations:** 1 Department of Materials Physics, Research School of Physics, Australian National University, Canberra ACT 2601, Australia; 2 Department of Electronic Materials Engineering, Research School of Physics, Australian National University, Canberra ACT 2601, Australia; 3 Research School of Chemistry, Australian National University, Canberra ACT 2601, Australia

**Keywords:** bioanalysis, biomarkers, biotechnology, diagnostics, nanotechnology

The concurrent evolution of nanotechnology and artificial intelligence offers exciting opportunities to develop new revolutionary technologies. One of the most promising advances resulting from the fusion of solid-state nanopores with artificial intelligence is label free biomarker quantification and analysis and disease detection which could have a transformative impact on the healthcare sector.

Solid state nanopores are nanoscale channels in a thin membrane material. These nanopores can be fabricated using different methods including but not limited to controlled breakdown, focused electron/ion-beam milling, track etch technology etc. Upon the application of a voltage across this membrane, when submerged in an electrolyte solution (usually a buffered salt solution such as LiCl, NaCl or KCl), ionic current flows through the nanopore. As biomolecules like DNA, RNA or proteins pass through the nanopore, they perturb this ionic flow, creating analyte-specific disruptions or signatures. While solid state nanopore sensing has been used for a variety of biomolecular analyses, one of the primary challenges has been in discerning between biomolecules of comparable sizes. Different proteins with similar size, for instance, could produce similar disruption patterns, making identification tricky. However, the signatures and the patterns which are generated by the translocation of different biomolecules can be analysed through artificial intelligence algorithms to determine the identity, size, shape and even conformation of the traversing molecules. Additionally, artificial intelligence algorithms can process vast amounts of data, find patterns and improve their accuracy over time without explicit programming.

In recent years, researchers have demonstrated a growing interest in utilizing solid-state and biological nanopore sensing along with artificial intelligence to detect viruses [^1-3^], DNA and RNA modifications [4] and sequences [5], and most recently proteins [6] in a label-free fashion. We have previously demonstrated how nanopore sensing combined with artificial intelligence can be utilized to identify similar-sized proteins without labelling – indistinguishable through pulse width and height centric analysis [6]. This highlights the significant potential of artificial intelligence for recognizing different biomolecules, offering almost real-time results for determining biomarker concentrations. The ability to ‘identify’ biomolecules from complex solutions offers ground-breaking potential for medical diagnostics. Once fully developed, the workflow for quantification of biomarkers using solid-state nanopore sensing assisted by artificial intelligence is rather simple. Bodily fluids such as blood, urine, cerebrospinal fluid (CSF) can be prefiltered and subsequently measured using a portable nanopore reader employing a target specific sized nanopore for the biomarker of interest. Different features of the signals will then be automatically analysed by artificial intelligence algorithms which provide information about biomarker identity and concentration.

Along with the evolution of artificial intelligence and nanopore sensing, a surge in research discovering new disease-specific biomarkers is emerging. Often a disease leads to systemic changes at the molecular and cellular level in patients. Depending on the pathological mechanisms contributing to diseases [7], e.g., inflammation, metabolic dysfunction, infection, ischemia, neuro/non-neuro degeneration, oxidative stress, apoptosis dysregulation etc., there can be an increase or decrease in one or multiple biomolecule levels in bodily fluids. For personalised measurement of these biomarker levels, new low-cost techniques that can be rolled out to point of care settings are highly desirable. Nanopore-based biomarker sensors could serve this purpose and pave the way for personalized healthcare. The ability of artificial intelligence algorithms to ‘learn’ from existing data allows it to differentiate between a myriad of molecular signatures, thus enhancing diagnostic accuracy. The inherent advantage of artificial intelligence is its iterative nature. As more data flows in, algorithms evolve, becoming more adept at making predictions and identifying anomalies. This continuous refinement ensures that diagnostic tools remain at the forefront of accuracy and reliability. The combination of solid-state nanopores and artificial intelligence promises to redefine how we detect, understand and subsequently treat diseases. Let's delve deeper into the various facets of this potential revolution.

Diseases like Alzheimer's, Multiple Sclerosis (MS) and Amyotrophic lateral sclerosis (ALS) are often detected only when they have reached advanced stages. Detecting protein aggregates or specific genetic markers using nanopore technology could signal the onset of these diseases much earlier, significantly increasing the ability for successful medical intervention. Artificial intelligence can help in correlating these biomarkers with disease progression. For example, ALS is a progressively fatal condition, usually resulting in a lifespan of 3–5 years after diagnosis. Early symptoms often overlap with other neurological disorders, starting with weakness in limbs, muscle twitching, or challenges in speech and breathing. Currently, there are no conclusive tests to diagnose ALS. Typically, it takes about 12 months from the first appearance of symptoms to diagnosis, relying heavily on excluding other potential causes. Distinguishing ALS from other neuromuscular diseases with similar symptoms can be challenging for clinicians. Several biomarkers that correspond to ALS also correspond to other diseases. For example, increased neurofilament light chain (NfL) levels could indicate Alzheimer's, Multiple Sclerosis and ALS. Solid state nanopore sensing coupled with artificial intelligence could provide a means for distinguishing between these diseases, as the algorithms can be used to detect multiple biomarkers at once, hence assisting clinicians toward faster diagnosis. An earlier diagnosis for such neurodegenerative diseases could mean quicker access to emerging treatments or importantly, a quicker exclusion of ALS as the cause of the symptoms.

Another use of artificial intelligence and nanopores is in the detection of viruses. The importance of rapid, low-cost, and regular screening of pathogens was essential during the COVID-19 pandemic. During such virus-related outbreaks, time is of the essence. Traditional culture methods can sometimes take days to identify pathogens. Nanopore devices, on the other hand, can detect pathogens in almost real-time at low concentrations. artificial intelligence can then classify these pathogens, guiding treatment and/or quarantine decisions. For example, *M. Taniguchi and colleagues* [1] have used solid state nanopores and artificial intelligence to detect SARS-CoV-2 from saliva.

Nanopore technology with the aid of artificial intelligence can also provide personalised healthcare. Nanopore sensing can profile lipids in blood, providing detailed analyses. Artificial intelligence can then correlate these profiles with cardiovascular risks, offering insights into preventive measures. For many diseases (cancer being one of the prominent ones), there's always a looming risk of recurrence post-treatment. Continuous monitoring using nanopore technology could detect molecular markers indicative of a relapse, ensuring immediate intervention. The future might even see wearable devices equipped with nanopore sensors continuously monitoring our health. artificial intelligence algorithms can provide real-time feedback, predicting potential health issues before they become severe. By combining data from nanopore devices with other health measures (like ECG, blood pressure), artificial intelligence could offer a holistic view of an individual's health, advancing preventive and personalized medicine. With the potential miniaturization and cost-reduction of nanopore devices, rural clinics and resource-limited regions could gain easier access to state-of-the-art diagnostic tools, bridging the gap in healthcare inequalities.

However, there are still many hurdles to overcome. In terms of membrane materials, there is a drive to invent nanopores in novel materials as well as nanopores of different shape for high signal to noise ratio, enhanced sensitivity and selective and/or specific detection of different biomolecules [8]. In general, the raw data from nanopore experiments is often replete with noise. Whereas high bandwidth measurements are important to get more information from the biomolecule translocation, such measurements often have more noise and result in large amounts of data (up to 150 MB/s) making it computationally expensive to analyse [6,9]. Advanced artificial intelligence algorithms are crucial to discern genuine molecular signals from noise as well as fast enough to analyse such a large amount of data. Further research is needed to refine these algorithms and ensure high-fidelity readings. Beyond the technological challenges, there's a broader need for the establishment of standard protocols, calibration methodologies and practices. Consistency in applying and integrating the technology into existing diagnostic systems is essential to ensure its widespread and effective use. Additionally, as with all emerging technologies, the regulatory landscape needs to be navigated, and concerns related to data privacy and security must be addressed. Together with advances in AI, the continuing improvement of nanopore experimental design, protocols and measurement hardware will still play a significant role in the outcome of this merger.

In conclusion, the convergence of nanopore sensing and artificial intelligence shows exciting future potential to redefine disease detection and biomarker analysis. While challenges remain, the future looks promising. However, collaborations, investments in research, and ethical considerations, are crucial for its success. Like all nascent technologies, it is difficult to predict how far and wide the use will spread but the rapid advancements in artificial intelligence and nanopore research suggest a trajectory of significant and far-reaching impact.
